# Exploring nature’s antidote: unveiling the inhibitory potential of selected medicinal plants from Kisumu, Kenya against venom from some snakes of medical significance in sub-Saharan Africa

**DOI:** 10.3389/fphar.2024.1369768

**Published:** 2024-04-12

**Authors:** Mitchel Okumu, James Mbaria, Joseph Gikunju, Paul Mbuthia, Vincent Madadi, Francis Ochola

**Affiliations:** ^1^ Department of Public Health, Pharmacology, and Toxicology, University of Nairobi, Nairobi, Kenya; ^2^ Department of Medical Laboratory Science, Jomo Kenyatta University of Agriculture and Technology, Nairobi, Kenya; ^3^ Department of Veterinary Pathology, Microbiology, and Parasitology, University of Nairobi, Nairobi, Kenya; ^4^ Department of Chemistry, School of Physical and Biological Sciences, University of Nairobi, Nairobi, Kenya; ^5^ Department of Pharmacology and Toxicology, Moi University, Eldoret, Kenya

**Keywords:** *Bitis arietans*, medicinal plants, Naja ashei, preclinical efficacy evaluation, Naja subfulva, snake venom, *Artemia salina* bioassay

## Abstract

**Background:** The present study investigated the efficacy of *Conyza bonariensis, Commiphora africana, Senna obtusifolia, Warburgia ugandensis, Vernonia glabra,* and *Zanthoxylum usambarense* against *Bitis arietans* venom (BAV), *Naja ashei* venom (NAV), and *Naja subfulva* venom (NSV).

**Methods:** 40 extracts and fractions were prepared using n-hexane, dichloromethane, ethyl acetate, and methanol. *In vitro* efficacy against snake venom phospholipase A_2_ (svPLA_2_) was determined in 96-well microtiter and agarose-egg yolk coagulation assays. *in vivo* efficacy against venom-induced cytotoxicity was determined using *Artemia salina*. Two commercial antivenoms were used for comparison.

**Results:** The 96-well microtiter assay revealed poor svPLA_2_ inhibition of BAV by antivenom (range: 20.76% ± 13.29% to 51.29% ± 3.26%) but strong inhibition (>90%) by dichloromethane and hexane fractions of *C. africana*, hexane and ethyl acetate extracts and fraction of *W. ugandensis*, dichloromethane fraction of *V. glabra*, and the methanol extract of *S. obtusifolia*. The methanol extract and fraction of *C. africana*, and the hexane extract of *Z. usambarense* strongly inhibited (>90%) svPLA_2_ activity in NAV. The hexane and ethyl acetate fractions of *V. glabra* and the dichloromethane, ethyl acetate, and methanol extracts of *C. africana* strongly inhibited (>90%) svPLA_2_ in NSV. The agarose egg yolk coagulation assay showed significant inhibition of BAV by the dichloromethane fraction of *C. africana* (EC_50_ = 3.51 ± 2.58 μg/mL), significant inhibition of NAV by the methanol fraction of *C. africana* (EC_50_ = 7.35 ± 1.800 μg/mL), and significant inhibition of NSV by the hexane extract of *V. glabra* (EC_50_ = 7.94 ± 1.50 μg/mL). All antivenoms were non-cytotoxic in *A. salina* but the methanol extract of *C. africana* and the hexane extracts of *V. glabra* and *Z. usambarense* were cytotoxic. The dichloromethane fraction of *C. africana* significantly neutralized BAV-induced cytotoxicity*,* the methanol fraction and extract of *C. africana* neutralized NAV-induced cytotoxicity, while the ethyl acetate extract of *V. glabra* significantly neutralized NSV-induced cytotoxicity. Glycosides, flavonoids, phenolics, and tannins were identified in the non-cytotoxic extracts/fractions.

**Conclusion:** These findings validate the local use of *C. africana* and *V. glabra* in snakebite but not *C. bonariensis, S. obtusifolia, W. ugandensis*, and *Z. usambarense.* Further work is needed to isolate pure compounds from the effective plants and identify their mechanisms of action.

## Introduction

An estimated 5 million people are bitten by snakes every year, about half of whom experience clinical illness, and up to 140,000 die from complications related to envenomation (Chippaux, 1998; Kasturiratne et al., 2008). Snakebites are prevalent among low-income individuals residing in rural, tropical areas with limited access to healthcare (Oliveira et al., 2023). Consequently, local people frequently rely on folk medicine, which includes the use of medicinal plants. Several such plants, including *C. bonariensis, C. africana, S. obtusifolia, Warburgia ugandensis, Vernonia glabra,* and *Zanthoxylum usambarense* have gained notoriety among the Luo people in Kisumu, Kenya, due to their putative anti-snake venom properties (Owuor et al., 2005; Owuor and Kisangau, 2006). These plants are known by the locals as “yadh asere” (*C. bonariensis*), “arupiny” (*C. africana*), “olusia” (*V. glabra*), “sogo” (*W. ugandensis*), and “roko” (*Z. usambarense*). They have widespread ethnomedicinal use locally including in snakebite and share phylogenetic relationships with plants previously reported as anti-snake bite remedies, e.g., *Senna siamea*, *Conyza sumatrensis*, and *Zanthoxylum chalybeum* (Owuor and Kisangau, 2006). Treatments include the use of cut, suck, and bind techniques, followed by the application of plant leaf and root poultices secured with bark or cloth strips (Owuor et al., 2005). However, there is a general concern about the efficacy and safety of alternative remedies in managing diseases (Puzari et al., 2022). Rigorous scientific scrutiny of these remedies is essential to determine the validity of the ethnomedicinal claims and to ensure the development of safe and efficacious interventions for snakebite victims (Puzari et al., 2022).

**FIGURE 1:**
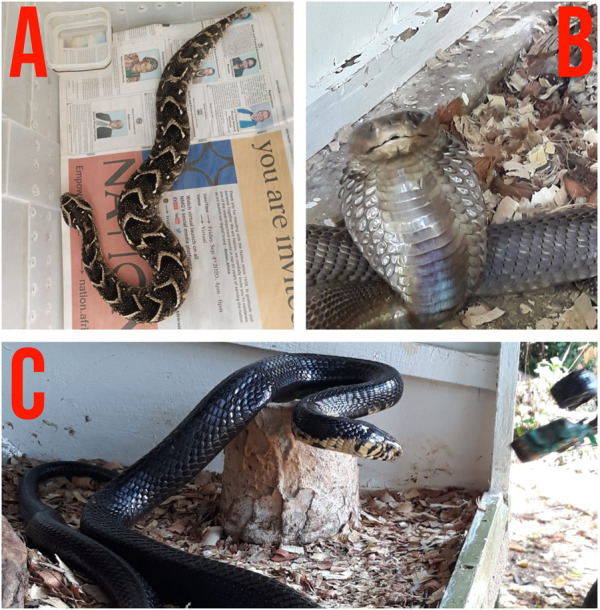
Photos of *Bitis arietans* **(A)**, *Naja ashei* **(B)**, and *Naja subfulva* **(C)**. Photos by Mitchel Okumu.

*B. arietans, N. ashei,* and *N. subfulva* are snakes of medical importance in sub-Saharan Africa (Calvete et al., 2007; Currier, 2012; Tasoulis and Isbister, 2017; Onyango, 2018; Okumu et al., 2020; Dyba et al., 2021) (Figure 1). Antivenom is the mainstay of treatment for envenomation by these snakes but is expensive, has limited availability, and does not sufficiently neutralize some key venom toxins, e.g., cytotoxins which cause dermonecrosis in snake bite victims. Medicinal plants are used to plug this gap, but they lack scientific validity. This study employed a combination of *in vitro* and *in vivo* methods to evaluate the antivenom properties of *C. bonariensis, C. africana, S. obtusifolia, W. ugandensis, V. glabra,* and *Z. usambarense* against *B. arietans*, *N. ashei*, and *N. subfulva* venoms.

## Materials and methods

### Collection and identification of medicinal plants

**FIGURE 2:**
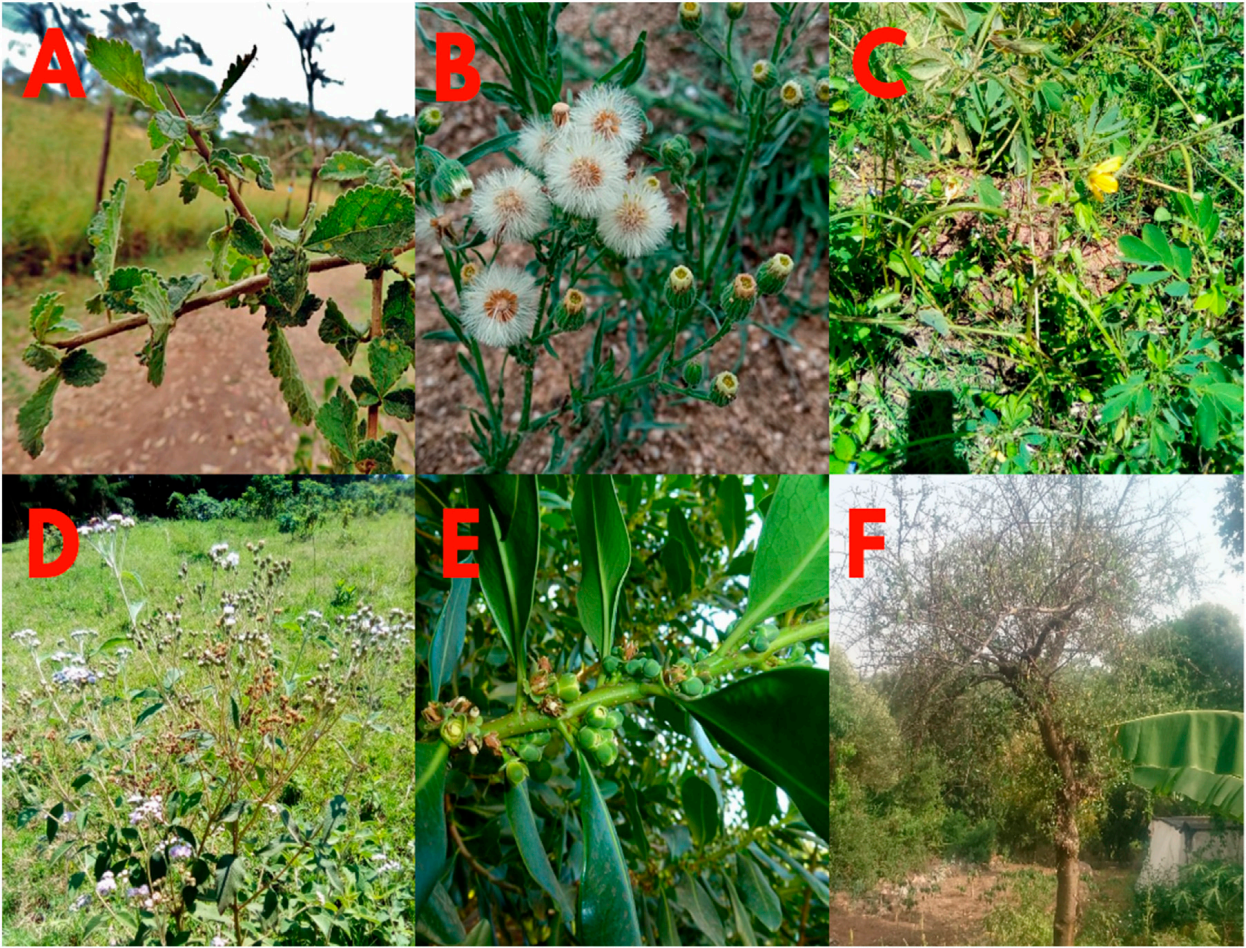
Photos of *Commiphora africana* (A. Rich) Engl **(A)**, *Conyza bonariensis* (L.)., Cronquist **(B)**, *Senna obtusifolia* (L.) Irwin and Barneby **(C)**, *Vernonia glabra* (Streetz) **(D)**, *Warburgia ugandensis* (Sprague) **(E)**, and *Zanthoxylum usambarense* (Engl.) Kokwaro **(F)** used in this study.

Plant materials were collected in November 2016 in Kisumu County. The East African Herbarium in Nairobi, Kenya identified and verified the plant specimens, as shown in (Supplementary Figure S1) (Supplementary Section). **REF NMK/BOT/CTX/1/2/1.** The selection of the plants was based on five factors: 1) their extensive local ethnopharmacological use in treating snakebites; 2) their evolutionary link to other plants used for the same purpose; 3) the findings of an Owuor and Kisangau survey on the use and practice of herbal medicine (Owuor and Kisangau, 2006), 4) the lack of published research outlining the plants’ bioactive ingredients, and 5) their availability for evaluation. An overview of the plants used in this study is as shown in (Figure 2).

### Preparation of plant material

After being cleaned to get rid of any dust that stuck to them, the plant materials were shade-dried and then ground into a powder using an electric mill (Retsch Grindomax, Germany).

### Chemical and reagents

n-hexane, dichloromethane, ethyl acetate, and methanol were purchased from Loba Chemie (India). Phosphate buffered saline (PBS) tablets, Calcium chloride, Fuchsin acid (Carbol Fuchsin), gallic acid, catechin, agarose, and rutin were bought from Sigma Aldrich (USA). Sodium carbonate, Folin-phenol reagent, Folin-Denis reagent, aluminum chloride, sodium hydroxide pellets, lead acetate, Sodium hydrogen phosphate, and picric acid were bought from FINAR (India). The antivenoms used in this study were manufactured in India and Mexico.

### Soxhlet extraction of the medicinal plants

Powdered plant materials were sequentially extracted by Soxhlet extraction using n-hexane, dichloromethane, ethyl acetate, and methanol and concentrated under reduced pressure at 40°C on a rotary evaporator (Stuart, Cole-Parmer-UK) (Janardhan et al., 2014). The percentage yield of the extracts was calculated as %w/w.

### Extraction of medicinal plants using a modified maceration technique

Powdered plant materials were separately mixed with methanol, macerated for 72 h, and concentrated at 40°C under reduced pressure on a rotary evaporator (Stuart, Cole-Parmer-UK). The methanol extracts were separated into four parts, distributed in de-ionized water, partitioned sequentially with n-hexane, dichloromethane, ethyl acetate, and concentrated under reduced pressure at 40°C on a rotary evaporator (Stuart, Cole-Parmer-UK) (Alsayari et al., 2018). The percentage yield of the extracts was calculated as %w/w.

### Ethics

The biosafety, animal care, and use committee of the University of Nairobi was consulted before the authors handled any experimental animal, as shown in Supplementary Figure S2 (Supplementary section) (REF BAUEC/2019/220).

### Snake venom

Nine specimens of the large brown spitting cobras (*N. ashei*), Eastern Forest cobras (*N. subfulva*) and puff adders (*B. arietans*) were collected in the wild and identified by a herpetologist at Bioken snake farm, Kenya. Venom was collected from these snakes using the beaker method, snap frozen, lyophilized (Labconco, USA), and kept as a powder at −20°C until it was reconstituted in phosphate buffered saline.

### Determination of the *in vitro* anti-snake venom phospholipase A_2_ activity of the prepared extracts

#### The 96-well microtiter plate assay

The methods of Iwanaga and Suzuki (Iwanaga and Suzuki, 1979) and Molander and colleagues (Molander et al., 2014) were used. 10 μL of a 10 μg/mL concentration of each of the venoms (in 0.1 M phosphate buffered saline) and 20 µL of a 100 μg/mL concentration of each of the prepared extracts were micro pipetted (Finnpipette, Thermo Fisher Scientific, USA) into 96-well microtiter plates (Costar^®^3590, USA) before 200 µL of a 1.1% egg yolk suspension in 0.1 M PBS adjusted to pH 8.1 and 0.2 mM CaCl_2_ was added to each well, and the absorbance of the mixtures was taken at 620 nm on a multi plate reader (Thermo Fisher Multiskan, USA). The plates were incubated (Memmert, Germany) at 37°C for 20 min and the absorbance measured again at 620 nm. svPLA_2_ activity was measured as the decrease in turbidity of the egg yolk suspension from 0 to 20 min. The inhibition of svPLA_2_ activity by the extract was expressed as percentage inhibition of enzymatic activity taking the absorbance of a well to which no venom was added as 100%. Extracts were tested in triplicate and antivenom was used as a positive reference.

#### The agarose-egg yolk coagulation assay

Extracts with >90% inhibition of the svPLA_2_ activity in the aforementioned assay were further evaluated in the agarose egg yolk coagulation assay described by Habermann and Hardt (Habermann and Hardt, 1972) as follows.

1. **Group I (Venom only group):** 10 µL of graded (0.5 μg/mL to 10.0 μg/mL) dilutions of venom only.
2. **Group II (Venom + extract/fraction mixture group):** Pre-incubated mixture of 10 μL of venom (0.5 μg/mL to 10.0 μg/mL) + 20 µL of a 100 μg/mL concentration of each of the extracts/fractions.
3. **Group III (Venom + antivenom):** Pre-incubated mixture of 10 μL of venom (0.5 μg/mL to 10.0 μg/mL) + 20 µL of a 100 μg/mL concentration of each of the antivenoms.

These mixtures were micro pipetted into 0.5 mm wells on an agarose-egg yolk medium and incubated (Memmert, Germany) at 50°C for 24 h. 10% Carbol Fuchsin was used to visualize the enzymatic halos in each group and the diameter of the enzymatic halos was measured using a digital vernier calliper (Rolson, United Kingdom) and expressed as the minimum phospholipase concentration (MPC) i.e., the least dose of venom which is responsible for an enzymatic halo of 10 mm in the case of BAV and 15 mm in the case of NAV and NSV.

### Cytotoxicity of the venoms, extracts, and antivenoms in *Artemia salina*

The *in vivo* toxicities of the extracts, venoms, and antivenoms were evaluated in *Artemia salina* according to the method described by Meyer *et al.* (1982) with modifications as described by Nguta *et al.* (2014). This was replicated in 5 different sample tubes for each venom, extract, or antivenom concentration. Physiological buffer saline (1 mL) was used as the negative control and vincristine sulphate was used as the positive control.

### Neutralization of *Artemia salina* venom-induced cytotoxicity by the extracts and antivenom

The WHO pre-incubation neutralization protocol was used and adjusted to *A. salina* (WHO, 2016). Varying doses of the extracts or antivenom (50 μg/mL, 100 μg/mL, 200 μg/mL, 400 μg/mL, and 800 μg/mL) were incubated (Memmert, Germany) with a 2LC_50_ dose of each of the venoms at 37°C for 30 min. The resulting mixtures were added to vials containing *A. salina* and the survivors were counted after 24, 48, and 72 h of exposure. The median effective concentration of the extracts was defined as the minimum amount of extract (in µL) required to neutralize 1 mg of venom.

### Initial screening of the extracts for phytochemicals

Standard methods were used for preliminary phytochemical screening of the extracts and fractions (Kokate et al., 2006; Evans, 2009; Kumar et al., 2013). The presence of alkaloids (dragendorrf’s test), anthraquinones, carboxylic acids, cardiac glycosides (keller-killiani test), flavonoids (alkaline reagent test), phenolics (Ferric chloride test), phytosterols, resins, saponins (foam test), tannins (Ferric chloride test), and terpenoids (Salkowski test) were investigated.

### Quantitative phytochemical composition

Total phenolics, flavonoids, glycosides, and tannins were estimated using a UV-VIS spectrophotometer (Spectronic 21-D, USA). Analytical grade gallic acid, catechin, and rutin were used as standards.

#### Determination of total phenolic content (TPC)

The method of Harnafi et al. was used (Harnafi et al., 2008). The extracts/fractions were mixed with 7.5% w/v Na_2_CO_3_ solution and 2.5 mL of Folin-Ciocalteau reagent (FINAR, India), and the absorbance was read at 765 nm on a UV-VIS spectrophotometer (Spectronic 21-D, USA) and a gallic acid standard curve was generated. The assay was performed in triplicate and the results were expressed as milligrams of Gallic acid equivalents per Gram of the dry plant material (mg.GAE.g^-1^).

#### Determination of total flavonoid content (TFC)

The method of Atanassova et al. was used (Atanassova et al., 2011). The extract/fractions were mixed with distilled water, 5% w/v sodium nitrite (NaNO_2_), 10% w/v aluminum chloride (AlCl_3_), and 1 M sodium hydroxide (NaOH), and the absorbance was read on a UV-VIS spectrophotometer (Spectronic 21-D, USA) at 510 nm. The flavonoid content was determined from a catechin standard curve. The assay was performed in triplicate and the results were calculated as milligrams of Catechin equivalents per Gram of the dry plant material (mg. CE. g^-1^).

#### Tannin content

The method of Amadi et al. was used (Amadi et al., 2004). The extracts/fractions were boiled gently for 1 h and mixed with 2.5 mL of Folin-Denis reagent, 5 mL of saturated Na_2_CO_3_ solution, and 25 mL of distilled water. The mixture was left to stand for 30 min in a water bath (Memmert, Germany) at 25°C and the absorbance was read on a UV-VIS spectrophotometer (Spectronic 21-D, USA) at 700 nm. The tannin content was determined from a tannic acid standard curve. The assay was performed in triplicate and the results were calculated as below:

$$\text{Tannic acid }\left(\text{mg}/100g\right)=\frac{C\times \text{extract}\;\text{volume}\times 100}{\text{Aliquot}\;\text{volume}\times \text{weight}\;\text{of}\;\text{sample}}$$

Where C is concentration of tannic acid read off the graph.

#### Cardiac glycoside content

The method described by Muhamad and Abubakar (Muhammad and Abubakar, 2016) was used. The extracts/fractions were mixed with distilled water, 12.5% lead acetate, 47% w/v Na_2_HPO_4_, and Baljet reagent (95 mL of 1% picric acid+5 mL of 10% NaOH). A blank titration was carried out using 10 mL distilled water and 10 mL Baljet reagent (95 mL of 1% picric acid+5 mL of 10% NaOH). This mixture was allowed to stand for 1 hour and the absorbance was read on a UV-VIS spectrophotometer (Spectronic 21-D, USA) at 495 nm. The percentage (%) of total glycosides present in extracts/fractions was calculated as % of total glycosides= (A×100)/77 g %. Where A = absorbance of samples.

### Data analysis

The effect of each of the extracts/fractions/antivenoms on the minimum phospholipase concentration of venom (s) was compared using one way-ANOVA and Dunnet’s multiple comparison test. The lethality of venoms, extracts, fractions, and antivenoms in *A. salina* and their capacity to neutralize venom-induced cytotoxicity in the same model was analyzed using probit regression analysis. Results on the phytochemical composition of the extracts/fractions were summarized in a table. *p < 0.05* was considered significant.

## Results

### The percentage yield of extracts

The percentage yield of the hexane root extract of *C. africana* prepared by the Soxhlet method was the lowest (0.23%), while the percentage yield of the dichloromethane leaf extract of *V. glabra* prepared by the maceration method was the highest (54.65%), as observed in (Supplementary Table S1).

### Information on the snakes whose venom was used in the study

Most of the snakes used in this study were sourced from the Watamu area in Kenya. (Supplementary Table S2) ***in vitro* microtiter well svPLA**_**2**_ **neutralization assay**.

The microtiter well assay revealed poor (<90%) anti-svPLA_2_ inhibition of BAV by the tested antivenoms (range: 20.76% ± 13.29% to 51.29% ± 3.26%) but potent (>90%) anti-svPLA_2_ inhibition of the venom by dichloromethane and hexane fractions of *C. africana* stem bark, hexane and ethyl acetate extracts and fraction of *W. ugandensis* leaves, dichloromethane fraction of *V. glabra* leaves, and the methanol extract of *S. obtusifolia* leaves.

>90% anti-svPLA_2_ inhibition was observed against NAV with the methanol extract and fraction of *C. africana* stem bark, the methanol extract from the *C. africana* bark, and the hexane extract of *Z. usambarense* leaves.

>90% anti-svPLA_2_ inhibition was noted against NSV with hexane and ethyl acetate fractions of *V. glabra* leaves and dichloromethane, ethyl acetate, and methanol extracts of *C. africana* bark (Table 1).

**TABLE 1:** The *in vitro* neutralization capacity of antivenom, extracts, and fractions of *Commiphora africana*, *Conyza bonariensis, Senna obtusifolia, Vernonia glabra, Warburgia ugandensis,* and *Zanthoxylum usambarense* against snake venom phospholipase A_2_.

			*Bitis arietans*		*Naja ashei*		*Naja subfulva*	
Plant species	Plant part	Solvent used	Soxhlet	Maceration	Soxhlet	Maceration	Soxhlet	Maceration
*Commiphora africana* (A. Rich.) Engl.	**Bark**	Hexane	No activity	24.24 ± 3.65	37.36 ± 6.29	No activity	8.33 ± 2.74	8.92 ± 1.58
		Dichloromethane	No activity	10.61 ± 2.98	39.06 ± 7.54	5.10 ± 1.34	21.58 ± 5.41	7.26 ± 2.34
		Ethyl acetate	No activity	62.12 ± 9.54	48.30 ± 8.76	No activity	27.14 ± 6.87	1.03 ± 0.89
		Methanol	No activity	50.00 ± 8.17	93.64 ± 2.55	82.80 ± 9.54	79.06 ± 9.23	54.98 ± 8.21
	**Stem bark**	Hexane	73.17 ± 5.28	96.18 ± 0.93	37.54 ± 6.87	5.21 ± 1.67	8.76 ± 2.34	5.18 ± 1.67
		Dichloromethane	14.63 ± 3.17	94.25 ± 3.21	33.96 ± 6.12	46.88 ± 8.32	7.48 ± 2.11	22.65 ± 5.89
		Ethyl acetate	21.95 ± 4.43	No activity	48.87 ± 8.43	79.17 ± 9.01	11.54 ± 3.42	23.3 ± 6.12
		Methanol	No activity	No activity	95.61 ± 0.28	95.55 ± 1.69	91.67 ± 5.32	83.17 ± 9.78
	**Roots**	Hexane	No activity	No activity	34.77 ± 7.32	No activity	20.85 ± 3.55	No activity
		Dichloromethane	No activity	No activity	33.20 ± 6.89	No activity	10.99 ± 2.87	16.59 ± 4.56
		Ethyl acetate	25.40 ± 6.72	No activity	44.20 ± 8.14	No activity	12.78 ± 2.94	No activity
		Methanol	7.94 ± 2.86	No activity	45.77 ± 8.26	21.82 ± 4.76	21.52 ± 5.98	No activity
*Conyza bonariensis* (L.) Cronquist	**Leaves**	Hexane	No activity	No activity	38.46 ± 7.68	35.76 ± 7.93	14.41 ± 7.63	No activity
		Dichloromethane	33.96 ± 7.14	10.34 ± 2.36	15.96 ± 4.23	No activity	8.30 ± 2.22	6.28 ± 2.01
		Ethyl acetate	41.51 ± 8.93	37.93 ± 7.84	35.77 ± 7.43	No activity	13.97 ± 2.28	No activity
		Methanol	37.74 ± 7.29	20.69 ± 4.71	23.08 ± 5.78	No activity	No activity	1.35 ± 0.78
*Senna obtusifolia* (L.) Irwin & Barneby	**Leaves**	Hexane	60.71 ± 10.47	5.00 ± 1.23	5.22 ± 1.78	13.56 ± 3.87	No activity	47.94 ± 8.09
		Dichloromethane	7.14 ± 2.14	2.50 ± 0.65	26.10 ± 5.56	No activity	28.19 ± 6.44	40.48 ± 7.56
		Ethyl acetate	No activity	No activity	36.14 ± 6.98	19.49 ± 4.98	67.55 ± 9.12	64.36 ± 9.32
		Methanol	94.33 ± 0.87	No activity	44.58 ± 8.32	7.06 ± 2.21	64.36 ± 9.99	50.75 ± 8.87
*Vernonia glabra* (Streetz) Vatke	**Leaves**	Hexane	76.19 ± 5.69	54.29 ± 8.45	17.36 ± 4.89	9.03 ± 2.87	97.39 ± 0.18	94.49 ± 3.76
		Dichloromethane	16.19 ± 3.81	93.33 ± 3.67	25.69 ± 5.21	No activity	99.42 ± 0.06	86.96 ± 9.23
		Ethyl acetate	78.10 ± 6.39	35.23 ± 6.89	No activity	34.03 ± 7.21	92.46 ± 1.76	91.88 ± 4.47
		Methanol	35.24 ± 7.57	37.14 ± 7.86	19.44 ± 4.76	No activity	96.81 ± 5.47	No activity
*Warburgia ugandensis* Sprague	**Leaves**	Hexane	40.74 ± 8.26	47.22 ± 8.63	43.97 ± 1.27	No activity	28.80 ± 6.67	14.89 ± 4.23
		Dichloromethane	28.70 ± 5.81	65.74 ± 9.86	33.62 ± 5.44	No activity	30.42 ± 7.21	24.60 ± 5.96
		Ethyl acetate	42.59 ± 9.72	40.74 ± 7.58	No activity	No activity	51.13 ± 8.65	27.18 ± 6.43
		Methanol	32.41 ± 6.25	50.00 ± 8.11	No activity	No activity	21.04 ± 5.69	38.51 ± 7.98
	**Leaf stalk**	Hexane	96.41 ± 0.22	5.00 ± 1.56	5.32 ± 1.45	14.41 ± 3.65	No activity	36.94 ± 7.23
		Dichloromethane	No activity	80.00 ± 9.47	30.04 ± 6.21	No activity	No activity	28.73 ± 6.12
		Ethyl acetate	92.69 ± 1.17	91.79 ± 4.15	8.37 ± 2.34	No activity	No activity	35.07 ± 7.34
		Methanol	37.50 ± 5.39	17.50 ± 3.14	13.31 ± 3.89	8.47 ± 2.54	No activity	25.37 ± 5.56
*Zanthoxylum usambarense* (Engl.) Kokwaro	**Leaves**	Hexane	No activity	No activity	58.23 ± 9.12	No activity	No activity	No activity
		Dichloromethane	No activity	No activity	19.28 ± 4.67	No activity	No activity	No activity
		Ethyl acetate	No activity	No activity	50.60 ± 8.78	No activity	No activity	No activity
		Methanol	No activity	No activity	29.72 ± 6.67	No activity	45.21 ± 8.78	No activity
	**Roots**	Hexane	No activity	No activity	93.25 ± 9.54	24.01 ± 5.43	No activity	15.50 ± 4.23
		Dichloromethane	No activity	No activity	85.23 ± 9.32	85.59 ± 9.23	No activity	22.48 ± 4.65
		Ethyl acetate	No activity	No activity	29.96 ± 6.43	31.36 ± 6.78	No activity	16.67 ± 5.89
		Methanol	No activity	No activity	33.76 ± 6.78	44.63 ± 8.41	No activity	25.78 ± 5.77
Vins bioproducts antivenom	**-**	-	51.29 ± 3.26		38.13 ± 4.99		20.76 ± 13.29	
Inoserp biopharma antivenom			38.96 ± 2.65		27.35 ± 10.70		25.76 ± 11.22	

#### *In vitro* agarose-egg yolk svPLA_2_ neutralization assay

**FIGURE 3:**
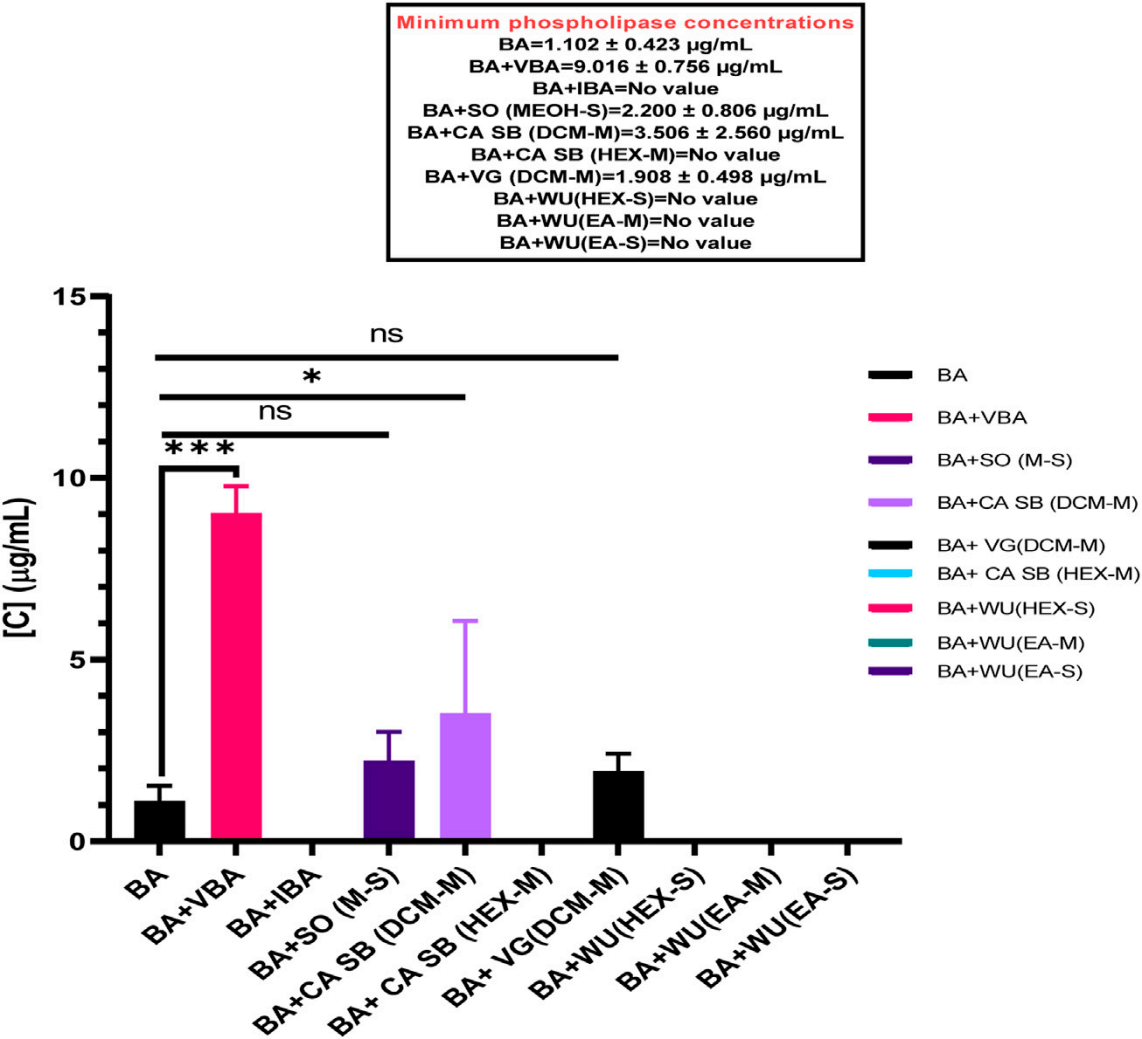
The effects of extracts, fractions, and antivenoms on the minimum phospholipase concentration of *Bitis arietans* venom. BA: *Bitis arietans*, VG (DCM-M): Dichloromethane fraction of *Vernonia glabra* leaves, CA SB (HEX-M): Hexane fraction of *Commiphora africana* stem bark, CA SB (DCM-M): Dichloromethane fraction of *Commiphora africana* stem bark. SO (MEOH-S): Methanol extract of *Senna obtusifolia* leaves, WU (HEX-S): Hexane extract of *Warburgia ugandensis* leaf stalk, WU (EA-M): Ethyl acetate fraction of *Warburgia ugandensis* leaf stalk, VBA: Vins bioproducts antivenom, IBA: Inoserp biopharma antivenom.

BAV had a minimum phospholipase concentration (MPC) of 1.102 ± 0.423 μg/mL. When separately incubated with various extracts, fractions, and antivenom, the MPC of the venom ranged from 1.908 ± 0.498 μg/mL to 9.016 ± 0.756 μg/mL. However, the only test substances that significantly inhibited *B. arietans* venom were Vins bioproducts antivenom, MPC = 9.016 ± 0.756 μg/mL (*p < 0.0001*) and the dichloromethane fraction of *C. africana* stem bark, MPC = 3.506 ± 2.560 μg/mL (*p = 0.0007*) (Figure 3).

**FIGURE 4:**
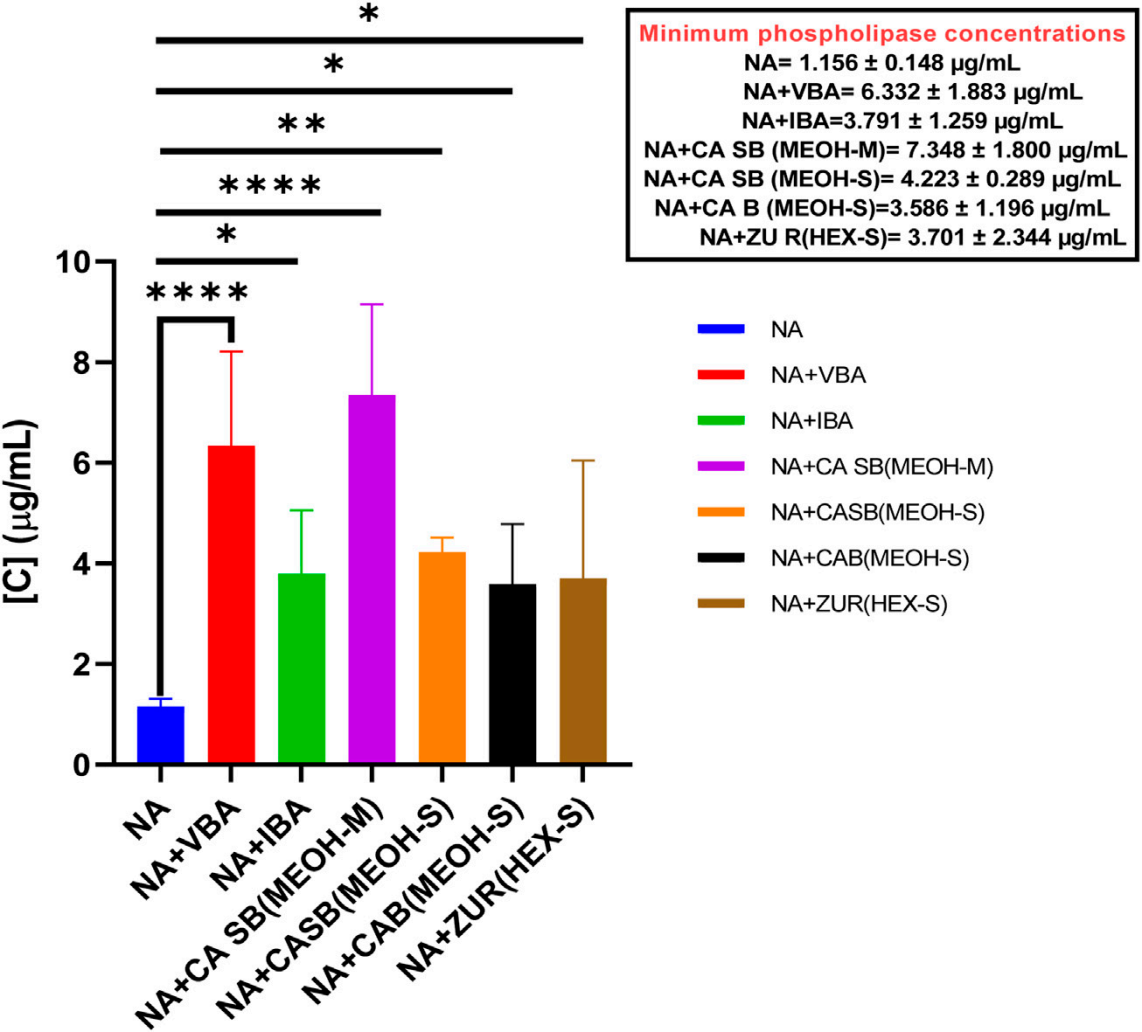
The effects of extracts, fractions, and antivenoms on the minimum phospholipase concentration of *Naja ashei* venom. NA: *Naja ashei*, CA B (MEOH-S): Methanol extract of *Commiphora africana* bark, ZU R (HEX-S): Hexane extract of *Zanthoxylum usambarense* root, CA SB (MEOH-S): Methanol extract of *Commiphora africana* stem bark, CA SB (MEOH-M): Methanol extract of *Commiphora africana* stem bark, VBA: Vins bioproducts antivenom, IBA: Inoserp biopharma antivenom.

NAV had an MPC of 1.156 ± 0.148 μg/mL. When separately incubated with various extracts, fractions, and antivenom, the MPC of the venom ranged from 3.586 ± 1.196 μg/mL to 7.348 ± 1.800 μg/mL. All the tested extracts, fractions, and antivenom significantly inhibited the phospholipase A_2_ activity of *N. ashei* including the methanol extract of *C. africana* bark, MPC = 3.586 ± 1.196 μg/mL (*p = 0.0343*), the hexane extract of *Z. usambarense* roots, MPC = 3.701 ± 2.344 μg/mL **(** *p = 0.0248*)*,* Inoserp biopharma antivenom, MPC = 3.791 ± 1.259 μg/mL (*p = 0.0191*)*,* the methanol extract of *C. africana* stem bark, MPC = 4.223 ± 0.289 μg/mL (*p = 0.0051*)*,* Vins bioproducts antivenom, MPC = 6.332 ± 1.883 μg/mL (*p < 0.001*)*,* and the methanol fraction of *C. africana* stem bark, MPC = 7.348 ± 1.800 μg/mL (*p < 0.0001*)*.* (Figure 4).

**FIGURE 5:**
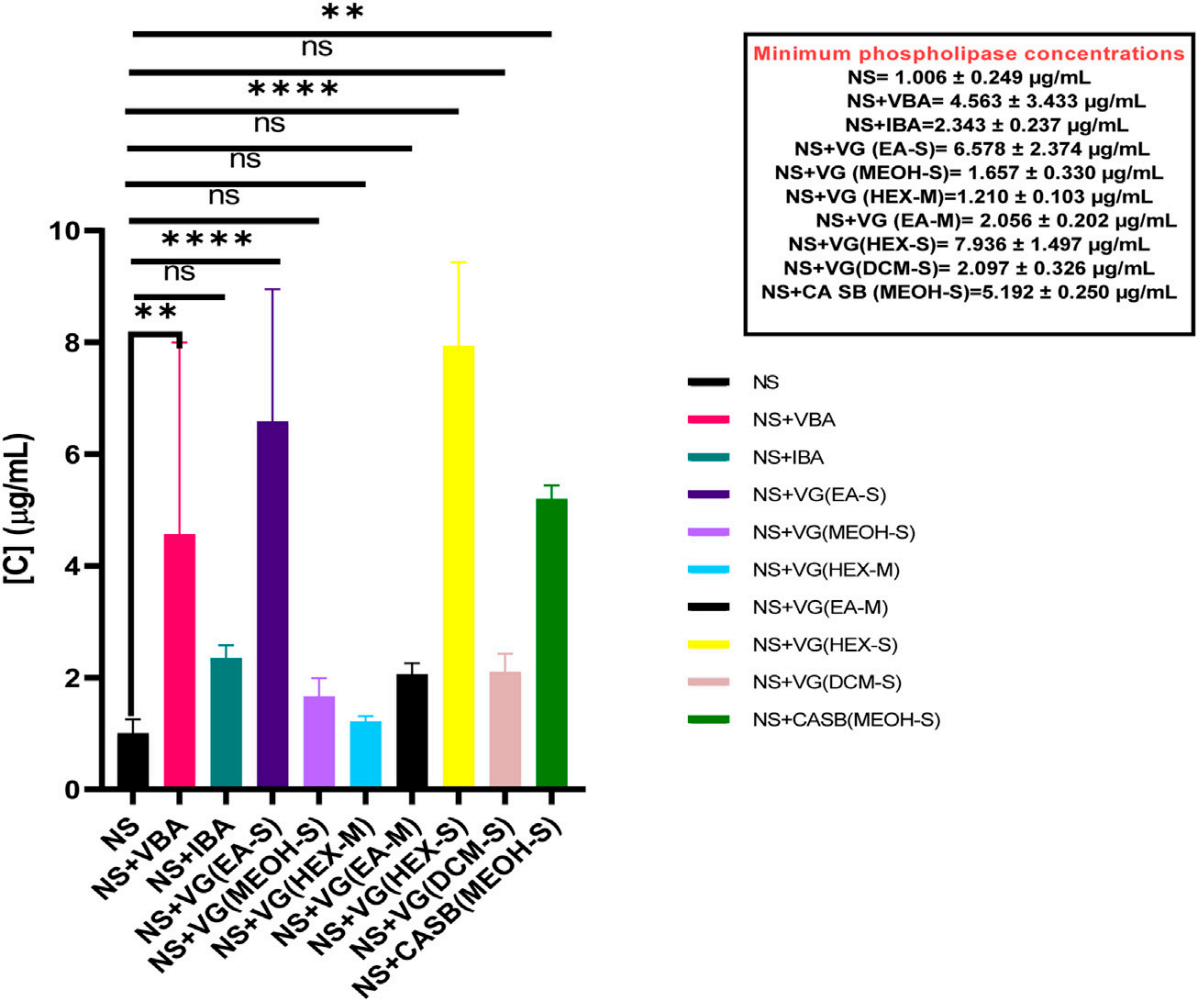
The effects of extracts, fractions, and antivenoms on the minimum phospholipase concentration of *Naja subfulva* venom in the snake venom phospholipase A_2_ agarose-egg yolk assay. NS: *Naja subfulva*, VG (DCM-S): Dichloromethane extract of Vernonia glabra leaves, VG (HEX-S): Hexane extract of Vernonia glabra leaves, VG (EA-M), Ethyl acetate fraction of *Vernonia glabra*, VG (HEX-M): Hexane fraction of *Vernonia glabra*, VG (MEOH-S): Methanol extract of *Vernonia glabra* leaves, VG (EA-S): Ethyl acetate extract of *Vernonia glabra* leaves, CA SB (MEOH-S): Methanol extract of *Commiphora africana* stem bark, VBA: Vins bioproducts antivenom, IA: Inoserp antivenom.

NSV venom had an MPC of 1.006 ± 0.249 μg/mL. When separately incubated with various extracts, fractions, and antivenom, the MPC of the venom ranged from 1.210 ± 0.103 μg/mL to 7.936 ± 1.497 μg/mL**.** However, the only test substances that significantly inhibited *Naja subfulva* venom were Vins bioproducts antivenom, MPC = 4.563 ± 3.433 μg/mL (*p = 0.0049*), the ethyl acetate extract of *V. glabra* leaves, MPC = 6.578 ± 2.374 μg/mL, the hexane extract of *V. glabra* leaves, MPC = 7.936 ± 1.497 μg/mL (*p < 0.0001*), and the methanol extract of *C. africana* stem bark, MPC = 5.192 ± 0.25 μg/mL (*p = 0.0022*) (Figure 5).

### Cytotoxicity of the extracts, fractions, and antivenom in *Artemia salina*

The methanol extract of *C. africana* stem bark, the hexane extracts of *V. glabra* leaves and *Z. usambarense* leaf stalk were cytotoxic to *A. salina* with LC_50_ values of 611.72 (251.06-3437.50) µg/mL, 0.04 μg/mL, and 31.54 (22.50-44.03) µg/mL respectively whereas the methanol stem bark fraction of *C. africana*, Vins bioproducts antivenom, and Inoserp antivenom were the least cytotoxic (Table 2).

**TABLE 2:** The cytotoxicity of antivenom, extracts, and fractions of *Commiphora africana, Vernonia glabra,* and *Zanthoxylum usambarense* in *Artemia salina*.

Description of the plant, solvent used, and the method of extraction				Number of dead *Artemia salina* per tested dose (n = 10)			LC_50_ (µg/mL)	Implication
Plant	Plant part	Solvent used	Method of extraction	10 μg/mL	100 μg/mL	1000 μg/mL		
*Commiphora africana* (A. Rich.) Engl.	Bark	Methanol	Soxhlet	00	00	06	3377.52 (No CI)	Non cytotoxic
	Stem bark	Dichloromethane	Maceration	00	00	03	6133.87 (No CI)	Non cytotoxic
	Stem bark	Methanol	Soxhlet	06	19	26	611.72 (251.06-3437.50)	Cytotoxic
	Stem bark	Methanol	Maceration	00	00	00	No death	Non cytotoxic
*Vernonia glabra* (Streetz) Vatke	Leaves	Hexane	Soxhlet	39	40	46	0.04 (No CI)	Cytotoxic
	Leaves	Ethyl acetate	Soxhlet	06	10	29	4049.78 (No CI)	Non cytotoxic
*Zanthoxylum usambarense* (Engl.) Kokwaro	Leaf stalk	Hexane	Soxhlet	07	43	50	31.54 (22.50-44.03)	Cytotoxic
Vins bio products antivenom	-	-	-	00	00	00	No death	Non cytotoxic
Inoserp biopharma antivenom	-	-	-	00	00	00	No death	Non cytotoxic
Vincristine	-	-	-	0	30	46	102.62 (No CI)	Cytotoxic
Sulphate (standard)								

LC_50_, Lethal concentration of the test substance responsible for the death of 50% of *Artemia salina* larvae; µg/mL; Micrograms per millilitre; CI, confidence interval.

### Qualitative phytochemical composition of extracts and fractions

Flavonoids, phenolics, glycosides, and tannins were found to be present in the dichloromethane and methanol fractions of *C. africana* stem bark, the methanol extract of *C. africana* bark, and the ethyl acetate extract of *V. glabra* leaves. However, alkaloids, carboxylic acids, phytosterols, and terpenoids were absent in the extracts and fractions (Table 3).

**TABLE 3:** Qualitative phytochemical composition of the extracts and fractions of *Commiphora africana* and *Vernonia glabra*.

Plant name	Solvent	Extraction method	Alkaloids	Anthraquinones	Carboxylic acid	Cardiac glycosides	Flavonoids	Phenolics	Phyto sterols	Resins	Saponins	Tannins	Terpenoids
*Commiphora africana* (A. Rich.) Engl. Stem bark	Dichloro methane	Maceration	-	+	-	+	+	+	-	-	-	+	-
	Methanol	Maceration	-	+	-	+	+	+	-	+	+	+	-
Bark	Methanol	Soxhlet	-	+	-	+	+	+	-	-	+	+	-
*Vernonia glabra* (Streetz) Vatke (leaves)	Ethyl Acetate	Soxhlet	-	-	-	+	+	+	-	-	+	+	-

^+^, Present; -: Absent.

### Quantitative phytochemical composition of the non-cytotoxic extracts and fractions

The ethyl acetate extract of *V. glabra* leaves had the highest glycoside (0.003%), total flavonoid (2.990 mg/g catechin equivalents), and tannic acid content (0.010%) while the methanol extract of *C. africana* stem bark had the highest phenolic content (2.180 mg/g gallic acid equivalents) (Table 4).

**TABLE 4:** Quantitative phytochemical composition of the extracts and fractions of *Commiphora africana* and *Vernonia glabra*.

Plant name	Solvent	Extraction method	Glycoside content (%)	Total phenolic content (mg/g of gallic acid equivalents)	Total flavonoid content (mg/g of catechin equivalents)	Tannic acid content (%)
*Commiphora africana* (A. Rich.) Engl. (stem bark)	Dichloromethane	Maceration	0.001	0.540	2.430	0.005
	Methanol	Maceration	0.001	1.100	0.600	0.008
	Methanol	Soxhlet	0.002	2.180	0.330	0.007
*Vernonia glabra* (Streetz) Vatke (leaves)	Ethyl Acetate	Soxhlet	0.003	0.490	2.990	0.010

### Neutralization of venom-induced cytotoxicity by extracts, fractions, and antivenom

The dichloromethane fraction of *C. africana* stem bark had an effective concentration of 336.12 ± 59.97 μg/mL against BAV-induced cytotoxicity in *A. salina.* The methanol extract of *C. africana* bark was the most effective against NAV-induced cytotoxicity in *A. salina* with an EC_50_ of 221.37 ± 30.33 μg/mL. The ethyl acetate extract of *V. glabra* leaves had an effective concentration of 329.39 ± 15.92 against NSV-induced cytotoxicity in *A. salina.* However, the test antivenoms were ineffective in neutralizing BAV*,* NAV and NSV-induced cytotoxicity in *A. salina* (Table 5).

**TABLE 5:** Neutralization of snake venom-induced cytotoxicity in *Artemia salina* by antivenom, extracts, and fractions of *Commiphora africana* and *Vernonia glabra*.

Venom	Inhibitor	Mortality per treatment						Neutralization efficacy of inhibitor
		2LC_50_ only	2LC_50_ + 50 μg/mL inhibitor	2LC_50_ + 100 μg/mL inhibitor	2LC_50_ + 200 μg/mL inhibitor	2LC_50_ + 400 μg/mL inhibitor	2LC_50_ + 800 μg/mL inhibitor	EC_50_
*Bitis arietans*	CA SB (DCM-M)	50	50	50	48	14	11	336.12 ± 59.97
	VBA	50	50	50	50	50	50	Ineffective
	IA	50	50	50	50	50	50	Ineffective
*Naja ashei*	CA SB (MEOH-M)	50	44	45	42	31	10	532.79 ± 169.04
	CA B (MEOH-S)	50	49	34	23	11	07	221.37 ± 30.33
	VBA	50	50	50	50	50	50	Ineffective
	IA	50	50	50	50	50	50	Ineffective
*Naja subfulva*	VG (EA-S)	50	50	49	40	18	08	329.39 ± 15.92
	VBA	50	50	50	50	50	50	Ineffective
	IA	50	50	50	50	50	50	Ineffective

LC_50_, Concentration of venom responsible for 50% mortality of *Artemia salina*; EC_50_, Concentration of extract/fraction or antivenom responsible for sparing 50% of *Artemia salina* from venom-induced death; CA SB (DCM-M), the dichloromethane fraction of *commiphora africana* stem bark; CA SB (MEOH-M), the methanol fraction of *Commiphora africana* stem bark; CA B (MEOH-S), the methanol extract of *Commiphora africana* bark; VG (EA-S), the ethyl acetate extract of *Vernonia glabra* leaves; VBA, vins bioproducts antivenom; IA, inoserp antivenom.

## Discussion

Snake venom phospholipases A_2_ (svPLA_2_) are enzymes which hydrolyze phospholipids and induce several pharmacological effects including edema, modulation of platelet aggregation, neurotoxicity, and myotoxicity (Six and Dennis, 2000; Kini, 2003; Pereanez et al., 2011). The present study observed that extracts and fractions of *C. africana, S. obtusifolia, V. glabra,* and *W. ugandensis* effectively neutralized *sv*PLA_2_s in BAV, NAV, and NSV. A similar study by Molander and colleagues evaluated the neutralization capacity of 226 extracts from 94 different plant species where it was reported that 11 water extracts and 28 ethanol extracts showed more than 90% inhibition against svPLA_2_ in *Bitis arietans* and *Naja nigricollis* venoms (Molander et al., 2014). These plants included *Lanea acida, Spondias mombin,* and *Capparis tometosa* (Molander et al., 2014).

Phytochemical analysis revealed that the extracts were rich in phenolics, tannins, saponins, and cardiac glycosides. Previous authors have demonstrated that phenolics, tannins, and saponins have antivenom properties (da Silva et al., 2007; Sia et al., 2011; de Moura et al., 2016; Salama et al., 2018; Liu et al., 2024). These antivenom properties were observed when *Saxifraga stolonifera, Rosmarinus officinalis, Plathymenia reticulata, Mimosa pudica,* and *Pentaclethra macroloba* were tested against venom from *Bothrops atrox, Cerastes,* and *Naja kaouthia* (da Silva et al., 2007; Sia et al., 2011; de Moura et al., 2016; Salama et al., 2018; Liu et al., 2024).

Cytotoxicity studies in *A. salina* revealed that some extracts of *V. glabra* leaves, *W. ugandensis* leaf stalk, and *C. africana* stem bark were cytotoxic to *A. salina*. Previous studies by Wanna, Karani, Anywar, Mwangi and their colleagues have shown that *V. glabra* was cytotoxic in *A. salina* (LC_50_ = 658 μg/mL) (Wanna et al., 2023), *W. ugandensis* was non-cytotoxic in Vero cells (CC_50_ of >250 μg/mL) (Karani et al., 2013) but cytotoxic to human glioblastoma cells (IC_50_ = 7.6 μg/mL) (Anywar et al., 2022) and *C. africana* was cytotoxic to Vero cells (CC_50_ > 20 μg/mL) (Mwangi et al., 2020). The compounds responsible for the toxicity of *V. glabra* and *Z. usambarense* have not been studied in detail but a study by Wairagu and colleagues established that cedrol, 9-octadecanoic acid-ethyl-ester, octadecadien-1-ol, citronellyl formate, n-hexadecenoic acid, and 1,2-dihydro-6-methoxy-naphthalene isolated from the dichloromethane crude fraction of *C. africana* resin were toxic to bedbugs (*Cimex lectularius*) (Wairagu et al., 2022). Moreover, E-resveratol 3-O-rutinoside isolated from the methanol fraction of *C. africana* stem bark was highly cytotoxic to breast (MCF-7), liver (HepG2), lung (A549), and prostate (PC3) cancer cell lines (Segun et al., 2019). In the case of *W. ugandensis*, compounds such as polygodial, warbuganal, ugandensolide, and mukaadial have been identified to be toxic against the maize weevil (*Sitophilus zeamais Motchulsky*) and the larger grain borer (*Prostephanus truncates Horn*) while compounds such as muzigadial have been found to be highly toxic to brine shrimp (*A. salina*) and *in vitro* trypanocidal activity against both drug-resistant and drug-sensitive trypanosome strains (Olila and Opuda-Asibo, 2001; Opiyo, 2020).

The *A. salina* model has been used to evaluate the cytotoxicity of medicinal plants (Nguta et al., 2011; Mwangi et al., 2015), environmental contaminants (Barahona and Sanchez-Fortun, 1999; Sanchez-Fortun and Barahona, 2009), and venom (Damotharan et al., 2015; Okumu et al., 2021). The present work was a continuation of our previous work where we investigated the capacity of two antivenoms to neutralize NAV-induced cytotoxicity in *A. salina* (Okumu et al., 2020). Moreover, we showed in another study that the *A. salina* model was a good surrogate for dermonecrosis in mice (Okumu et al., 2021). The present study established that some extracts and fractions of *C. africana* were effective in prolonging the survival of *A. salina* exposed to NAV. Isa and colleagues in a previous research reported that the crude methanol extract and fraction of *C. africana* dose-dependently neutralized *N. nigricollis* envenomation in mice (Isa et al., 2022). Abdullahi et al. reported the anti-snake venom properties of a *C. africana* related plant, i.e., *Commiphora pedunculata* against *N. nigricollis* venom (Abdullahi et al., 2017). While this study has highlighted the capacity of the prepared extracts to neutralize key effects of medically important sub-Saharan snakes, it did not evaluate the capacity of the extracts/fractions to neutralize other key toxins in the studied snake venoms including protease, hyaluronidase, and neurotoxins (3FTx’s). Moreover, further work is needed to understand the identity of the compounds responsible for the observed extract/fraction induced cytotoxicity in *A. salina.*

## Conclusion

These findings validate the local use of *C. africana* and *V. glabra* in snakebite envenomation and provide a basis for further work aimed at isolating pure compounds from these plants and identifying their mechanism of action. However, *C. bonariensis, S. obtusifolia, W. ugandensis*, and *Z. usambarense* use in snakebite is limited by poor efficacy and cytotoxicity.

## Data Availability

The raw data supporting the conclusion of this article will be made available by the authors, without undue reservation.

## Abbreviations

CaCl_2_, Calcium Chloride; CHCl3, Chloroform; ELISA, Enzyme Linked Immunosorbent Assay; EC_50_, Effective concentration of extract/antivenom that spares 50% of *A. salina* from death; FECl_3_, Ferric Chloride; H_2_SO_4_, Sulphuric acid, HCl, Hydrochloric acid; LC_50_, Lethal concentration that kills 50% of *A. salina*, Na_2_CO_3_, Sodium Carbonate, UV-VIS, Ultraviolet and visible, mg. GAE.g^-1^, milligrams of gallic acid equivalents per Gram, mg. CE. g^-1^, milligrams of catechin equivalents per Gram; MPC, Minimum phospholipase concentration; µL, Microliter; µg/mL, Microgram per millilitre; mM, millimoles; NaOH: sodium hydroxide; Na_2_WO_4_, Sodium tungstate, H_3_PO_4_, Phosphoric acid; SvPLA_2_, Snake venom phospholipase A_2_; WHO, World Health Organization.
