# Supplementary material for: Exploring nature’s antidote: unveiling the inhibitory potential of selected medicinal plants from Kisumu, Kenya against venom from some snakes of medical significance in sub-Saharan Africa
Source: Front Pharmacol. 2024 Apr 12;15:1369768. doi: 10.3389/fphar.2024.1369768 (PMC11045943; doi:10.3389/fphar.2024.1369768)

**Supplementary section**

**Table S1:** Plant name, part of the plant used, and yield of the prepared extracts.

| **Details of the medicinal plants** | | | **Yield w/w (%)** | | |
| --- | --- | --- | --- | --- | --- |
| **Plant name** | **Family** | **Part of the plant used** | **Extracts** | **Soxhlet** | **Maceration** |
| *Commiphora africana*   1. Rich) Engl. | Burseraceae | Stem bark | Hexane  Dichloromethane  Ethyl acetate  Methanol | 0.73  1.40  2.10  13.23 | 4.92  17.72  13.45  4.06 |
|  |  | Bark | Hexane  Dichloromethane  Ethyl acetate  Methanol | 0.97  1.50  0.73  13.00 | 2.71  6.94  18.10  4.42 |
|  |  | Roots | Hexane  Dichloromethane  Ethyl acetate  Methanol | 0.23  1.97  0.57  7.17 | 14.49  24.15  25.12  1.38 |
| *Conyza bonariensis*  (L.) Cronquist | Asteraceae | Leaves | Hexane  Dichloromethane  Ethyl acetate  Methanol | 1.13  2.20  1.73  13.20 | 14.43  22.68  30.93  0.95 |
| *Senna obtusifolia*  (L.) Irwin and Barneby | Fabaceae | Leaves | Hexane  Dichloromethane  Ethyl acetate  Methanol | 1.10  3.43  2.43  14.17 | 28.94  47.29  8.47  5.67 |
| *Vernonia glabra* (Streetz) Vatke | Asteraceae | Leaves | Hexane  Dichloromethane  Ethyl acetate  Methanol | 1.97  3.77  3.90  13.73 | 43.84  54.65  32.43  2.22 |
| *Warburgia ugandensis*  Sprague | Canellaceae | Leaves | Hexane  Dichloromethane  Ethyl acetate  Methanol | 2.07  6.47  2.40  19.07 | 16.18  17.53  23.37  2.97 |
|  |  | Leaf stalk | Hexane  Dichloromethane  Ethyl acetate  Methanol | 0.73  1.90  1.63  12.9 | 5.68  10.09  12.62  2.11 |
| *Zanthoxylum usambarense*  (Engl.) Kokwaro |  | Leaves | Hexane  Dichloromethane  Ethyl acetate  Methanol | 1.70  5.37  2.63  12.67 | 12.09  22.73  23.22  2.76 |
|  |  | Roots | Hexane  Dichloromethane  Ethyl acetate  Methanol | 1.27  3.97  1.17  4.90 | 8.29  23.31  31.09  2.57 |

**Table S2:** Gender age, location of capture, and reference number of the snakes whose venom was used in this study.

| **Species name** | **Common name** | **Reference number** | **Age** | **Gender** | **Location of capture** |
| --- | --- | --- | --- | --- | --- |
| *Bitis*  *arietans* | Puff  adder | BK17190  BK17193  BK18081 | Adult  Adult  Adult | Male  Female  Female | Watamu  Kizingo  Arabuko Sokoke |
| *Naja*  *ashei* | Large brown spitting cobra | BK16189  BK16648  BK17678 | Adult  Adult  Adult | Female  Female  Male | Watamu  Kilifi  Watamu |
| *Naja*  *subfulva* | Forest  cobra | BK10293  BK17954  BK18019 | Adult  Adult  Adult | Male  Male  Female | Kakamega  Busia  Nandi |

**Figure S1:** Document from the East African Herbarium at the National Museums of Kenya showing the identity of the studied medicinal plants (REF NMK/BOT/CTX/1/2/1).


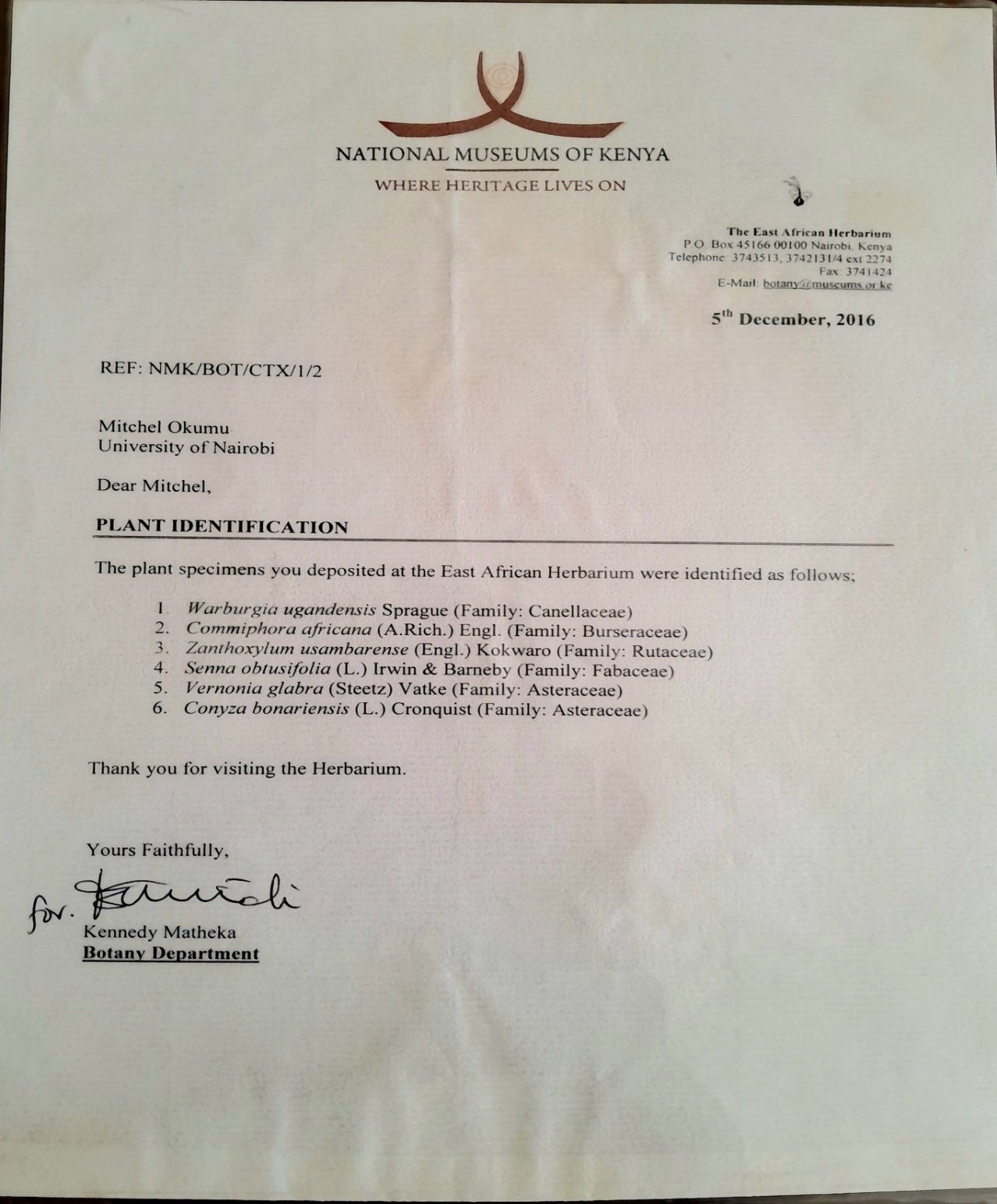


**Figure S2:** Ethical approval document from the Biosafety Animal Use and Ethics Committee at the University of Nairobi (REF BAUEC/2019/2020).


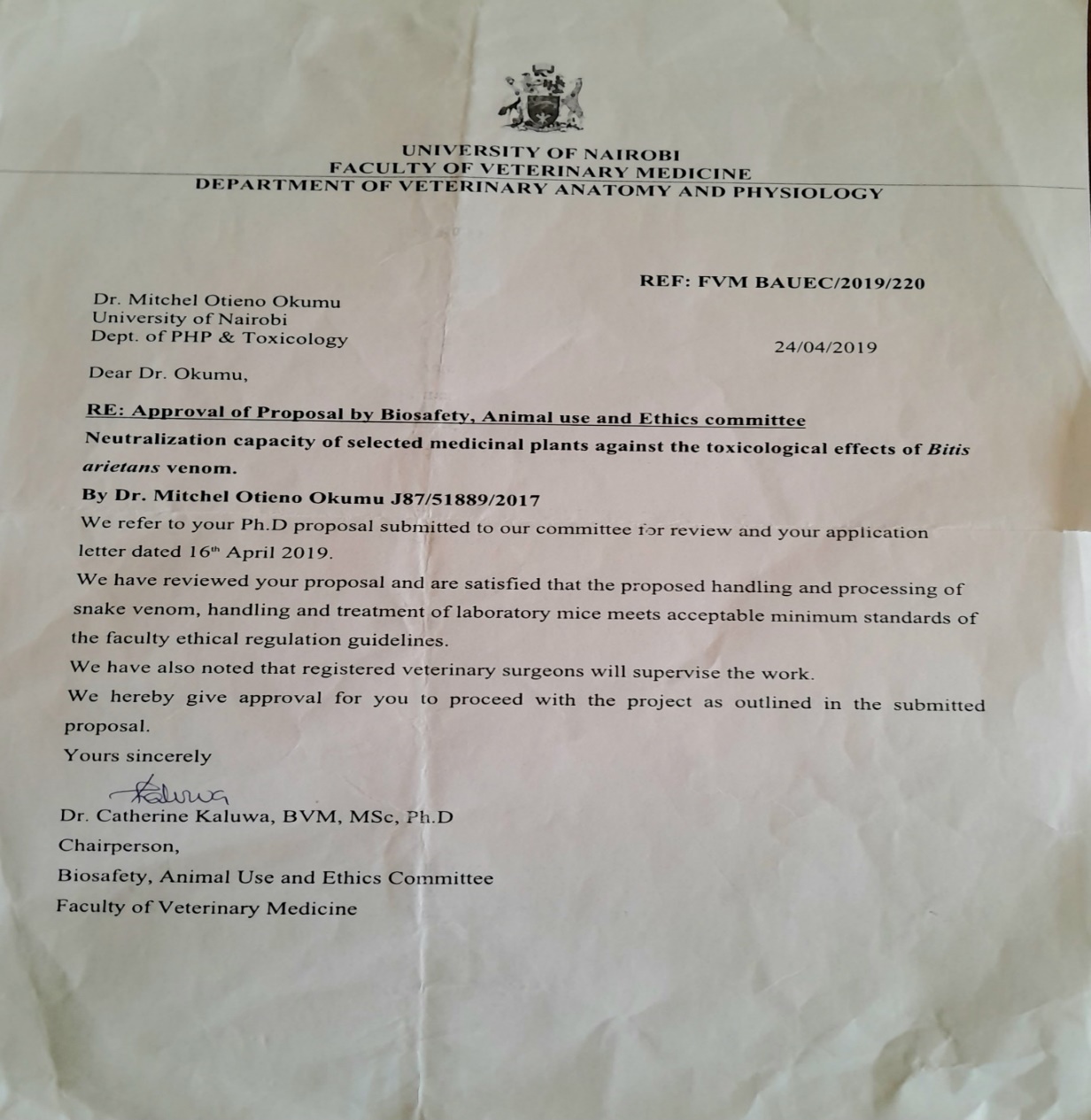

Supplement: Supplementary file 1 [file DataSheet1.docx]
